# Effect of Food Endotoxin on Infant Health

**DOI:** 10.3390/toxins13050298

**Published:** 2021-04-22

**Authors:** Haoming Wu, Yang Wang, Huiying Li, Lu Meng, Nan Zheng, Jiaqi Wang

**Affiliations:** 1 State Key Laboratory of Animal Nutrition, Institute of Animal Sciences, Chinese Academy of Agricultural Sciences, Beijing 100193, China; wuhaoming@caas.cn (H.W.); lihuiying@caas.cn (H.L.); menglu@caas.cn (L.M.); zhengnan@caas.cn (N.Z.); 2 Laboratory of Quality and Safety Risk Assessment for Dairy Products of Ministry of Agriculture and Rural Affairs, Institute of Animal Sciences, Chinese Academy of Agricultural Sciences, Beijing 100193, China; 3 Key Laboratory of Quality & Safety Control for Milk and Dairy Products of Ministry of Agriculture and Rural Affairs, Institute of Animal Sciences, Chinese Academy of Agricultural Sciences, Beijing 100193, China; 4 State Key Laboratory of Membrane Biology, School of Life Sciences, Tsinghua University-Peking University Joint Center for Life Sciences, Tsinghua University, Beijing 100084, China; wangyang881229@mail.tsinghua.edu.cn

**Keywords:** endotoxin, infant formula, infant health, alkaline phosphatase

## Abstract

Endotoxin is a complex molecule derived from the outer membrane of Gram-negative bacteria, and it has strong thermal stability. The processing of infant food can kill pathogenic bacteria but cannot remove endotoxin. Because the intestinal structure of infants is not fully developed, residual endotoxin poses a threat to their health by damaging the intestinal flora and inducing intestinal inflammation, obesity, and sepsis, among others. This paper discusses the sources and contents of endotoxin in infant food and methods for preventing endotoxin from harming infants. However, there is no clear evidence that endotoxin levels in infant food cause significant immune symptoms or even diseases in infants. However, in order to improve the safety level of infant food and reduce the endotoxin content, this issue should not be ignored. The purpose of this review is to provide a theoretical basis for manufacturers and consumers to understand the possible harm of endotoxin content in infant formula milk powder and to explore how to reduce its level in infant formula milk powder. Generally, producers should focus on cleaning the milk source, securing the cold chain, avoiding long-distance transportation, and shortening the storage time of raw milk to reduce the level of bacteria and endotoxin. After production and processing, the endotoxin content should be measured as an important index to test the quality of infant formula milk powder so as to provide high-quality infant products for the healthy growth of newborns.

## 1. Introduction

In the early stage of human life, the intestinal immune organs are not yet fully developed [1,2]. Breast milk provides children with rich nutrition and immunity protection, and supports the healthy growth of infants. For a variety of reasons, infants may not be able to obtain enough milk, which must then be supplemented with infant formula. To ensure the safety of food, food processors are used to kill microorganisms in raw food materials by means of heat treatment. However, killing microorganisms does not guarantee the absolute safety of infant food, due to endotoxin, lipoteichoic acid, peptidoglycan, and teichoic acid mannose. The endotoxin structure on the surface of Gram-negative bacteria can remain in food due to its strong thermal stability [3,4]. The residual endotoxin may have adverse effects on the immune development and intestinal health of infants. Of course, it is well known that, to date, no serious diseases have been reported in infants due to endotoxin contamination of food. However, it is undeniable that, for the healthy growth of infants and children, the presence of endotoxin in infant food and methods to reduce its content are worth discussing.

Immune stimulation by endotoxin in infants is not inevitable. Many studies on endotoxin and its effects on the body’s immune response have included the inhibitory effect of food additives on immune stimulation by this molecule. For example, many heat-sensitive substances exist in raw milk and are inactivated due to thermal sterilization. These include alkaline phosphatase, which has endotoxin-neutralizing ability, and lactoferrin, which can inhibit endotoxin toxicity [3,5,6]. In addition, researchers have found that probiotic oligosaccharides in breast milk can facilitate the proliferation and fermentation of probiotics in infants and young children. These probiotics (including lactic acid bacteria and *bifidobacteria*) play a positive role in the maintenance of intestinal homeostasis and the body’s resistance to endotoxin [4,7]. Controlling the content, source, and storage method of bacteria in raw milk will effectively reduce the content of endotoxin in the product.

This paper compiles the literature on the content of endotoxin in infant food, the possible harm of endotoxin to infants, and food additives or methods that can prevent or treat endotoxin contamination in order to improve the quality and safety of infant food, reduce unnecessary foodborne stress in infants, and suggest courses of action that will support the healthy growth of infants and young children.

## 2. Biological Activity of Endotoxin

Lipopolysaccharide (LPS) is the main molecule in the outer membrane of bacteria, and it can be released into the host in large quantities during infection [5,6]. At present, there is no conventional analysis or current laws/regulations to limit the concentration of endotoxin in foods in any country in the world, but many studies have shown that the content of endotoxin in infant food is very high. Therefore, it is necessary to further study its impact on health. In fact, endotoxin has been found in several foods, and it has shown resistance to cooking and low pH, and can stimulate inflammatory signals [8,9,10]. Studies have found that oral endotoxin can cause and spread small intestinal inflammation and destroy tight junction function [11,12,13]. Endotoxin concentration is usually expressed in ng/mL, where 1 ng/mL endotoxin is about 10–15 EU/mL [14,15]. A plasma endotoxin level of 1.0 ng/mL (0–15 EU/mL) is considered to have physiological effects. Intravenous injection of 4 ng/kg body weight of LPS has been shown to reduce blood pressure in volunteers when the concentration reaches 10 ng/mL (100–150 EU/mL). In addition, oral administration of 300 ng/mL (3000–4500 EU/mL) has been found to increase intestinal permeability in mice [16]. However, endotoxin from different Gram-negative bacteria, and even those stored under different conditions, can induce specific immune responses [17], which makes comparative studies difficult.

The standard LPS molecule has a three-part structure, including lipid A, a hydrophobic component that anchors LPS to the bacterial outer membrane; the core oligosaccharide, which helps lipid A maintain the integrity of outer membrane; and a polymer composed of repeated oligosaccharide units connected to the core and in contact with the external environment, called the O-antigen polysaccharide, or O-antigen [18]. Endotoxin molecules containing only lipid A and the core are often referred to as “rough” and known as lipooligosaccharides, while intact LPS capped by the O-antigen is called “smooth”. When bacteria enter the extracellular space, endotoxin plays a key role in the host–pathogen interaction of the innate immune system [19,20,21]. In the endotoxin structure, the relatively conservative lipid region (lipid A) is the bioactive part, which can induce a differential immune inflammatory response and even lead to septic shock [22,23]. Lipid A consists of 1,4′-diphosphate glucosamine disaccharide with six fatty acids and a straight chain length of 12–14 methyl(ene) units. Other lipid A-like species differ in the number, length, and composition of the attached fatty acids, as well as in the degree of phosphorylation and the number and type of substituted phosphate ligands. For example, Bacteroides fragilis (BF-LPS) lipid A is penta-acylated and monophosphorylated and contains branched chain fatty acids with a length of 15–17 methyl(ene) units; deviations from the standard lipid A structure are known to have a profound impact on the innate immune response of the host [20,24,25,26,27,28].

Lipid A expressed in typical endotoxin (*Escherichia coli* and most intestinal bacteria) consists of two amide bonds and two ester bonds, acyl and hydroxyl acyl chains of double phosphorylated glucosamine disaccharide. There are usually different acyloxy acyl chains that produce penta-acyl or hex-acyl lipid A. This is the main molecular lipid A species in most wild-type intestinal bacteria [29,30]. Endotoxin can activate Toll-like receptors (TLRs), which play an important role in activating the innate immune system of the host. These receptors activate the innate immune system by detecting Damage-Associated Molecular Patterns (DAMPs) or pathogen-associated molecular patterns (PAMPs), which are different from molecules belonging to the host organism but not inherent microorganisms or microbial secretions [25]. LPS stimulates multi-protein Toll-like receptor 4 (TLR4) by forming TLR4-MD-2-LPS-type complexes, thus activating downstream signaling cascades and transcription factors such as NF-κB and Interferon regulatory factor (IRF). In turn, transcription factors guide the production of various immune cells [31]. This can cause strong pathological reactions, including fever, hypotension, dyspnea syndrome, intravascular coagulation, and LPS shock. However, the structure of LPS from different bacteria varies, which affects the recognition by TLR4 and regulates different immune response pathways [32,33]. Changes in the structural arrangement of lipid A (such as a decrease in the charge number or acetyl chain number, or a change in distribution or saturation) lead to a significant reduction in toxicity. For example, the synthetic precursor tetra-alkyl lipid A is described as a non-endotoxin molecule and has been proposed as an antagonist of the hex-acyl endotoxin LPS [29,34]. The immunogenicity of lipid A from different microbial sources also varies. The lipid A extracted from *Escherichia coli* contains two phosphate groups and six acyl chain structures. The lipopolysaccharide from *Escherichia coli* secretes a large amount of necrosis factor κB (NF-κB)-dependent cytokines, such as interleukin-10 (IL-10), tumor necrosis factor-alpha (TNF-α), IL-1b, and IL-6a in primary Peripheral blood mononuclear cell (PBMCs), and it can induce cells to synthesize a large number of TLR4 and NF-κB responses; however, lipid A extracted from *B. dorei* has two ionic structures with one phosphate group and four and five acyl chains. However, the lipopolysaccharide from *B. dorei* cannot stimulate cells to induce TLR4 signaling or NF-κB response signals, regardless of the dose [35].

## 3. Endotoxin in Infant Food

Breast milk is the best source of nutrition for newborns. However, infant formula becomes a necessary substitute for breast milk when the infant is unable to obtain sufficient amounts for various reasons. The quality and safety of infant formula milk (IFM) directly affects the healthy development of infants, especially premature infants. High-temperature sterilization can effectively kill pathogenic bacteria in raw milk and ensure the safety of milk powder microorganisms. Unfortunately, endotoxin synthesized by Gram-negative bacteria in raw milk is stable at 250 °C and remains in the milk after heat treatment [36]. It may threaten the health of infants with incomplete immune development [37]. In Table 1, we have summarized the results of endotoxin tests performed on raw milk and dairy products in recent years and calculated the oral dose received from 100 g or 100 mL of milk. In any case, at present, there is no clear evidence that endotoxin in infant food can cause severe immune symptoms or diseases in infants. In a milk powder survey, infant formula studies from seven countries and 31 brands found endotoxin levels ranging from 40 EU/g to 55,000 EU/g [37]. This is equivalent to 0.067–91.67 µg/kg endotoxin per day (calculated according to an infant weight of 6 kg and 100 g of milk powder per day, for example). The endotoxin level in milk and milk products is still far from the dose that can cause a severe immune reaction (250 µg/kg) [38,39,40], which suggests that the endotoxin content in milk powder cannot cause disease in infants and young children. However, heat tolerance leads to endotoxin remaining in dairy products. The higher the number of Gram-negative bacteria in raw milk, the higher the content of LPS in the products [41]. Therefore, although there is a possibility that microbial contamination occurs during processing, the endotoxin content of the product may partly reflect the level of microorganisms in the raw milk of infant formula.

Gram-negative bacteria in raw milk are the main sources of endotoxin in dairy products. Studies have found a positive correlation between the amount of *E. coli* added to raw milk and the endotoxin content in the milk product [41]. According to the food and drug administration (FDA), the total bacterial count in raw milk should not be higher than 5,000,000 cells/mL [47]; according to the standards of the EU, Australia, and New Zealand, the total number of bacteria in raw milk should not exceed 500,000 cells/mL [48]. China’s national raw milk standard requires that the total bacterial count in raw milk be less than 20,000,000 cells/mL [49]. For pathogenic microorganisms, such as *Salmonella*, coliforms, Enterobacteriaceae, and *Listeria monocytogenes*, there are corresponding requirements and regulations in different countries [47,48,49]. In the composition standard of additives for infant formula milk powder, the limits of endotoxin in the fermentation process and adequate production of 2′-O-fucosyllactose and lacto-N-neotetraose (LNnT) are <10 EU/mg [50,51,52]. However, there is currently no standard for the limit of endotoxin in infant formula milk powder. Monitoring the total number of bacteria in raw milk and dairy products is insufficient. If raw milk contains a large number of Gram-negative bacteria or the storage time of raw milk is prolonged, endotoxin will remain in processed dairy products, although there is no clear evidence that endotoxin in dairy products can significantly cause infant discomfort or even disease. However, the endotoxin content can also partly reveal the level of microbial contamination in the processing of raw milk and dairy products. In order to improve the quality of infant food, it is necessary to establish a limit test for endotoxin in infant formula.

There are many kinds of active complexing agents in raw milk that form complexes with endotoxin to inhibit its toxicity. Heat-labile molecules in milk include immunoglobulin, lactoferrin, alkaline phosphatase, complement factor, lysozyme, and high mobility group protein 1 (HMBG 1) [43]. These thermally unstable molecules form stable structures with endotoxin, thus reducing its toxicity [53]. However, the biological activity and endotoxin-binding activity of these proteins are decreased or even lost after heat treatment. These complexes lose their activity at high temperatures, leading to the release of endotoxin. During processing, increasing the heat treatment intensity and changing the sterilization method will affect the content of endotoxin in the product [54].

## 4. Effect of Endotoxin on Infants

### 4.1. Intestinal Immune Structure in Infants

**Figure 1:**
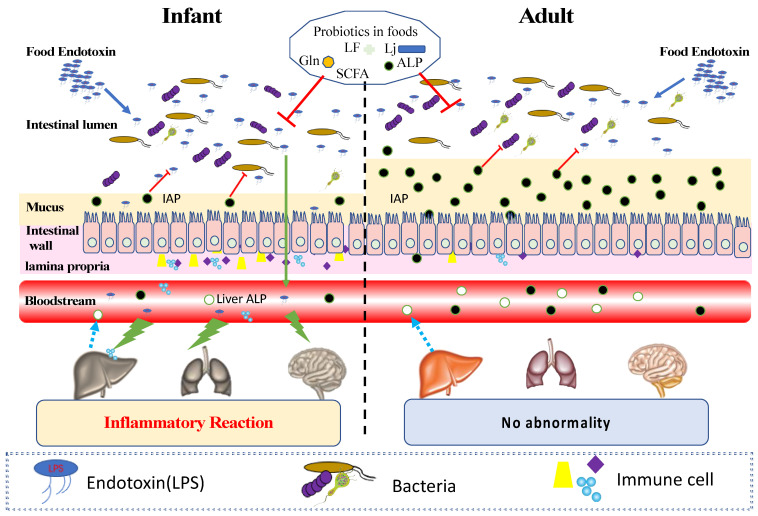
The intestinal barrier is not fully developed in infancy, and the thinner mucosa and intestinal structure make it easier for endotoxin to enter the blood. When endotoxin enters the circulation, it may stimulate inflammation in the liver, lung, and brain. However, for healthy adults, endotoxin can be removed by alkaline phosphatase reaction in intestinal mucosa to maintain health. Prebiotics and probiotics in food can help the intestinal tract resist the proliferation of microorganisms and the invasion of endotoxin.

After birth, the intestinal barrier is constantly exposed to potentially harmful environmental factors, including food ingredients and bacterial endotoxin [1]. The innate immune barrier is of great help in protecting the infant body, preventing bacterial invasion, and promoting immune homeostasis (Figure 1). At birth, the intestinal tissue structure is not mature and needs to develop gradually; during this stage, tolerance to harmful environmental substances and food is weak. Despite the presence of goblet cells, the expression of mucoprotein (MUC2, MUC3, and MUC5AC) in the intestinal tract of newborns is very low compared with adults, which leads to thinner mucosa and greater vulnerability to environmental or food-induced damage [2]. The microbial diversity in the gut of a newborn is low: it is only one-third of that of adults. When intestinal barrier function and immune homeostasis are impaired (intestinal dysfunction), an inflammatory state may develop and affect overall health [55]. Such physiological characteristics make the intestinal tract of infants more vulnerable to the invasion of foreign toxins and can even lead to disease [56].

In contrast to the traditional concept, bacterial colonization occurs in the intestinal tract of human newborns [57]. When infants are exposed to microorganisms in the extrauterine environment, their intestinal tract is rapidly colonized by external bacteria, which leads to the further development of the intestinal microbiota [56,58,59]. Different delivery methods, feeding methods, maternal diet structure, pet exposure, and antibiotic use will significantly affect the composition of intestinal flora and the immune development of infants [60,61,62,63,64]. Infant feeding characteristics, such as the nature of the food (breast milk and/or formula feeding) and the start time of feeding, can significantly affect the colonization and development of intestinal bacteria in infants [65]. Nutrition in food is an important factor in the colonization of intestinal flora [66]. In infancy, the intestinal bacterial colonization of the newborn will determine whether the baby can maintain their health while growing up. Compared with infant formula and soybean milk, breastfed infants have better neurodevelopment characteristics, motor development, and language development [67]. The colonization of intestinal flora in breastfed infants is significantly different from that in formula-fed infants [68]. There are more probiotics in the intestinal tract of breastfed infants, such as *Bifidobacterium infantis*, *Lactobacillus acidophilus,* and *Bacteroides fragilis*, and higher bacterial diversity is found in the intestinal tract of breastfed infants [69]. Breastfed infants have more immune protection genes than formula-fed infants [68]. The colonization of these probiotics is related to the oligosaccharide content in breast milk [70]. Oligosaccharides are fermented by bacteria in the infant’s colon, resulting in the proliferation of a large number of probiotics (such as *Bifidobacterium infantis*) [71]. The fermentation of these bacteria results in an acidic environment in the intestine and increases the production of short-chain fatty acids to promote the early development of mucosal immunity, increases the expression of tight junction proteins, and provides anti-inflammatory effects [72].

### 4.2. Effects on Infant Health

In general, the human gut contains about 10^12^ CFU/g bacterial cells, which maintain a delicate balance with the intestinal epithelium [73]. In normal human intestine, nearly 1 g of endotoxin (about 10^10^ EU) is prevented from entering the blood through the intestinal epithelium under conventional conditions [74,75]. However, for infants and young children with immature immune function, and the elderly with immune function degradation, the accumulation of intestinal endotoxin is a great threat. In Table 2, the immune responses induced by oral administration and intraperitoneal injection of endotoxin in mice are summarized. It can be seen that when the oral endotoxin concentration reaches 250 µg/kg, mice develop enteritis and express inflammatory-related factors [38]. During the growth of infants, the intake of infant milk powder is 500 g/kg by mouth. For an infant weight of 6 kg, the monthly consumption of milk powder is 3000 g; that is, the daily intake of an infant is about 100 g. Taking the endotoxin content in milk powder as an example, the daily intake of endotoxin in 6 kg infants was 1,000,000 EU/day. Ten EU of endotoxin is about 1 ng, so the daily intake of endotoxin is about 100 µg/day. The daily oral endotoxin concentration in infants is about 16.7 µg/kg, which is about one-tenth of the level that produces a significant immune response. For infants with a weak immune system, the endotoxin content in food should not be ignored.

There is very little evidence that endotoxin can cause illness in the normal healthy population [85]. Indeed, oral LPS can even treat allergies and lifestyle-related diseases [86]. Endotoxin in the intestines of healthy people is considered to be healthy and harmless. Many studies have suggested that oral endotoxin does not pose a threat to the health of animals and actually has a probiotic effect [87,88]. In milk research, it was found that although raw milk had a high endotoxin concentration (Table 1), endotoxin in milk reduced the incidence of allergic diseases caused by endotoxin in aerosols [89]. On the contrary, when the proliferation of Gram-negative bacteria in the intestine leads to a large increase in endotoxin, and if the barrier function of the intestine is destroyed, endotoxin in the intestine will be released, leading to severe inflammation [90,91]. Higher levels of endotoxin can be detected in the blood of obese patients with type 2 diabetes, non-alcoholic fatty liver disease (NAFLD), pancreatitis, amyotrophic lateral sclerosis, and Alzheimer’s disease [92,93,94,95,96]. The oral intake of endotoxin can increase the content of endotoxin in the blood, which leads to an immune response (Table 2). It was found that the concentration of endotoxin in the serum of mice increased by 1.5 times after treatment with 300 μg/kg endotoxin by oral gavage for 2 hours, which also caused the mice to exhibit anxious behavior [40]. The intestine, liver, and lung cytokine-induced neutrophil chemoattractant 1(CINC-1) concentrations in mice fed 250–500 μg of endotoxin orally increased by about four times (equivalent to human IL-8); plasma and lung TNF-α concentrations also increased significantly. The oral administration of endotoxin can significantly increase the levels of IL-1β, IL-6, IL-10, IL-18, CINC-1, and TNF-α in lung tissue [39]. Microscopically, the number of crypts and branches in the epithelial cells of ileal villi in pups treated with endotoxin was significantly increased, and the mucosal structure was distorted. The mucosal changes induced by endotoxin were consistent with those before necrosis [39]. In newborns, necrotizing enterocolitis, bronchopulmonary dysplasia, intraventricular hemorrhage, and intraventricular leukomalacia are associated with proinflammatory cytokines. This may be related to the synthesis and secretion of proinflammatory cytokines induced by endotoxin invasion. On the other hand, endotoxin in blood can directly induce neuroinflammatory reaction through the blood–brain barrier (BBB) [97]. LPS in prenatal and neonatal blood can increase the sensitivity of the brain to hypoxia and ischemic events, causing brain damage [98]. So far, there is not enough evidence that oral LPS can cause serious diseases, but it still needs to be paid enough attention.

Endotoxin in the intestine can cause serious inflammation when it enters the blood. In addition, food ingredients can help endotoxin enter the blood of consumers. The lipid components in food can improve the permeability of LPS in the gut and allow the food and LPS in the intestine to enter the blood [99]. A high-fat diet (HFD) has been shown to lead to metabolic endotoxemia in animals and humans [100,101]. It was found that endotoxin and chylous particle complexes could enter mesenteric lymph and circulate in vivo. A high-fat diet leads to excess chylous particle synthesis, which leads to chylophilia and eventually causes systemic inflammation [102]. On the other hand, an HFD was also found to cause local intestinal inflammation [103]. Thus, it causes systemic and local inflammation, which leads to the overexpression of inflammatory cytokines, an increase in intestinal permeability, the acceleration of endotoxin transfer, and a vicious cycle of endotoxemia [104,105,106]. Mice lacking Toll-like receptor 4 (TLR4) (endotoxin receptor) were significantly resistant to developing characteristics of HFD-induced metabolic syndrome, such as obesity and insulin resistance [107]. The cause of metabolic syndrome caused by an HFD is related to metabolic endotoxemia [108]. An HFD is associated with imbalances in the composition and quantity of normal microorganisms in the gut (malnutrition), leading to barrier dysfunction, followed by the transfer of LPS to the systemic circulation [109]. An increasing number of studies have indicated that metabolic endotoxemia is the pathogenesis of metabolic syndrome. When the endotoxin concentration in circulating blood is more than 2–3 times the normal level, it is defined as endotoxemia. Researchers have used the serum levels of TNF-α, IL-1, and IL-6 as evidence of metabolic endotoxemia [110]. On the other hand, LPS stimulation can also lead to the slow development of infant immune function and other problems. Compared with breastfeeding, the growth of the intestine of cubs fed LPS was decreased [38]. Similar to human beings, the effects of endotoxin exposure on the body have also been found in mammalian studies. Short term prenatal exposure to LPS in amniotic membrane can cause acute neonatal intestinal and pulmonary inflammation in premature pigs and is prone to systemic inflammation after delivery [111]. When 40 mg of each animal was added to the feed of adult pigs, the intestinal inflammation of pigs appeared, and even led to systemic endotoxemia [112]. In contrast, some studies believe that when pigs are repeatedly fed the same endotoxin, the sensitivity of pigs to this kind of endotoxin will be reduced [85]. Therefore, for newborns, endotoxin exposure may cause immune response and immune memory. It is biased to judge the advantages and disadvantages of endotoxin alone. Of course, if we can remove the toxicity of endotoxin and make the infant produce immune memory, it will be the best choice for infants

## 5. Prevention and Treatment

### 5.1. Killed Allies: Alkaline Phosphatase

Similar to breast milk, raw milk consumed without prior treatment has also been shown to reduce the risk of allergic diseases in many studies [6,113,114,115,116]. In a mouse model, raw milk inhibited allergic asthma caused by house aerosols and food allergies caused by ovalbumin (OVA) [117]. Due to the possible contamination by pathogens, including *Salmonella*, regulatory authorities do not encourage the consumption of raw milk [118]. Although the risk of certified raw milk produced according to strict hygiene and microbiological standards is considered low, raw milk will never be associated with zero risk. Therefore, milk is processed for commercial purposes. The shelf life of milk can be prolonged by homogenization and heat treatment. Unfortunately, milk processing reduces the protective effect of milk against asthma and allergies [3,5,6]. Milk processing greatly changes the composition of milk and has a significant impact on its fat content and heat-sensitive components. The hot processing method changes the content of n-3 polyunsaturated fatty acids in milk [3]. At the same time, heat damage to whey protein components and alkaline glutaminase in milk increases the risk of allergies [6].

Alkaline phosphatase (ALP) is a zinc-containing dimer with a molecular weight of 86,000 Da. Each subunit contains 429 amino acids, and the two subunits are connected by four cysteine residues. A large amount of alkaline phosphatase (>150 U/L) has been found in raw milk. In milk production, alkaline phosphatase is more heat-resistant than *Mycobacterium paratuberculosis*. ALP is regarded as the standard of milk product sterilization when test results for alkaline phosphatase in milk are negative. ALP is an excellent antidote to endotoxin. ALP mitigates the toxicity of endotoxin by decomposing the phosphate bond component in lipid A. As a result, endotoxin cannot stimulate the internal immune environment of consumers, but can prevent and treat diseases such as inflammation and asthma.

ALP can be synthesized in human organs; it can be self-synthesized in the liver, kidney, bone (ALPL), bile duct, intestinal mucosa (ALPI), and placenta (ALPP) with clear structural homology and functional similarity [119]. High alkaline phosphatase activity can be detected in the uterus of pregnant women. High alkaline phosphatase activity in the gut of full-term newborns, combined with high alkaline phosphatase activity in breast milk during the first few days of life, provides sufficient capacity to detoxify bacterial endotoxin that initially colonizes the infant intestine. Alkaline phosphatase activity is low in the preterm gut and in the absence of early postpartum breastfeeding, which increases the risk of excessive inflammation and Necrotizing enterocolitis (NEC) development. Therefore, prophylactic supplementation of ALP in preterm infants may be an effective treatment to prevent NEC.

To evaluate alkaline phosphate levels in the blood as immune function matures, a survey was conducted on 167,625 children. ALP activity in boys reached the highest value at 12–13 years of age and decreased to the lowest value at 18–19 years of age. ALP activity in girls reached the highest value at 10–11 years of age and gradually decreased to the lowest value at 17–18 years of age [120]. Intestinal Alkaline phosphatase (IAP), as a natural intestinal brush boundary enzyme, plays a key role in the aging process by maintaining the dynamic balance of intestinal flora, protecting intestinal barrier function, and reducing inflammation. Alkaline phosphatase can prevent liver injury caused by a high-fat diet. ALP prevented HFD-induced liver weight gain and protected mice from HFD-induced increases in liver enzymes, namely, aspartate aminotransferase (AST), gamma glutamyl transferase (GGT), and alanine aminotransferase (ALT). Alkaline phosphatase can protect the host intestinal microbial immunity [121] and can be used as an effective supplement to prevent endotoxemia and protect the host from metabolic syndrome. Alkaline phosphatase can reduce the symptoms of elevated endotoxin in the blood caused by corn oil and prevent inflammation and intestinal permeability changes caused by an HFD. In the same study, it was found that the secretion of alkaline phosphatase was increased in rats fed with a high-fat diet [122]. It has been suggested that the body also adopts a way of secreting alkaline phosphatase with an HFD. Alkaline phosphatase was found to be mainly expressed in proximal intestinal epithelial cells and to then diffuse into the intestinal cavity and then into systemic circulation after secretion [123]. The intestinal flora of IAP knockout mice was significantly different from that of wild-type mice [124]. In a study that used a zebrafish model, it was confirmed that there was a clear correlation between alkaline phosphatase and intestinal flora. The expression of alkaline phosphatase only began when zebrafish were exposed to a bacterial environment, but it was not detected under sterile conditions [125]. It is worth noting that the content of alkaline phosphatase seems to be related to immune capacity. Endogenous alkaline phosphatase is lower in infancy and old age when the immune level is lower. In this period, the intestinal tract is more vulnerable to endotoxin shock, which indicates that a “low level of IAP” may be a factor in the induction of metabolic syndrome [126]. However, in the infant stage, due to the heat sterilization process, there is no alkaline phosphatase in infant milk powder. Therefore, Chinese people who rarely eat raw and cold food have almost no exogenous alkaline phosphatase, except for after birth when they are exposed to breast milk. This may be the reason for the difference in infant immune function.

### 5.2. Raw Milk Management

Milk is a high-quality medium for bacteria. Many kinds of bacteria can proliferate and grow in milk. However, microorganisms are abundant in pastures where cows live. Milking methods, livestock feed, environmental conditions [127,128], the environment of the barn, and the equipment used may contaminate raw milk [129]. Different cow management conditions, such as outdoor feeding [130], animal location [131], and lactation stage [132], all affect the microbial composition of milk. Therefore, reasonable planning and management of the cow feeding environment, lactation, and pasture can effectively reduce microbial contamination in raw milk and improve the quality and safety of dairy products.

In pastures, raw milk is rapidly cooled after being collected and temporarily stored in milk tanks at 4–8 °C. After that, it is transferred to a milk tank truck and transported to the dairy processing plant in the cold chain. During this process, raw milk may be stored at a low temperature for 24–72 h. Psychrophilic bacteria in raw milk, including *Pseudomonas* and *Acinetobacter*, can proliferate in large quantities [133,134,135]. These Gram-negative bacteria can live in a low-temperature storage environment, release protease and lipase to reduce the quality of milk, and secrete extensive levels of endotoxin into dairy products during sterilization. Similarly, when infant formula milk powder is brewed, the milk may be contaminated by proliferating psychrophilic bacteria [54]. Therefore, shortening the transportation and storage time of raw milk will help to reduce the content of microorganisms. Low microbial counts in raw milk can effectively reduce the content of heat-resistant protease, heat-resistant lipase, and endotoxin in milk and improve the quality of infant food.

### 5.3. Probiotics

Many studies have shown that the balance of intestinal flora determines health-related conditions in the host, including enteritis, obesity, diabetes mellitus, and even brain/nerve-related diseases (Table 3). Studies have found that endotoxin can affect the function of the central nervous system; for example, LPS released by a large number of *Bacteroides* can cause systemic inflammation and even lead to Alzheimer’s disease (AD) [136]. Studies have found that a variety of probiotics in the gut play a “guard” role and effectively lower the invasion of endotoxin. *Lactobacillus johnsonii* (LJ) can effectively restore disordered intestinal microflora, increase the expression of tight junction proteins in Caco-2 cells, inhibit the activation of NF-κB, reduce the levels of intestinal microflora and LPS in the blood, and alleviate memory impairment and colitis caused by 2,4,6-trinitrobenzenesulfonic acid (TNBS) and *Escherichia coli* (EC) [4]. *Bifidobacterium* can inhibit the expression of TLR2 and TLR4 in the intestine and prevent TLR-mediated inflammation. It plays a protective role by inhibiting inflammation and preventing the penetration of pathogenic bacteria in patients with inflammatory bowel disease [7]. The addition of probiotics may contribute to the intestinal microbiological health of infants and young children.

## 6. Conclusions

This paper introduces and summarizes the sources, structure, possible influence, and prevention of endotoxin in food. When the content of endotoxin in infant food is too high, it may threaten the health of infants. In order to reduce endotoxin levels in infant food, we should reduce microbial pollution in pastures, reduce the number of bacteria in raw milk, shorten the transportation time of raw milk, reduce heat damage in the process of sterilization, and retain more prebiotics in the final product. At the same time, we hope to call on the government and relevant departments to formulate a standard limit of endotoxin in infant food, which can reduce the possible threat posed by endotoxin to the healthy growth of infants and support their healthy development.

## Figures and Tables

**Table 1:** Endotoxin content in food.

	Model	Region	Sample Size/Dose	100 mL/Day	References
Raw milk	Raw and UHT milk ^1^	Belgium	0.19–5800 EU/mL	19–580,000 EU	[42]
	Milk tanks	Hungary	3–6144 EU/mL	300–614,400 EU	[43]
	Raw milk	Pullman, Wash	10–10^10^ EU/mL	1000–10^12^ EU	[44]
	Raw milk	Germany Austria Switzerland Finland France	1–10^7^ EU/mL	100–10^9^ EU	[10]
	Raw milk	Iran	0.063–0.25 EU/mL	6.3–25 EU	[41]
	Low SCC ^2^ raw milk (health)	Japan	0.27–2.16 EU/mL	27–216 EU	[45]
	High SCC raw milk (health)		0.28–42.0 EU/mL	28–4200 EU	
Milk production	Processed shop milk	Hungary	60–240 EU/mL	600-24,000 EU	[43]
	Powdered instant formula milk	South Africa, Holland, Spain, Switzerland, USA, Belgium, Ireland, Slovenia UK	40–55,000 EU/g	400–550,000 EU	[37]
Other infant food products	Commercial infant cereal-based products	Sweden	1400–24,200 EU/g	14,000–242,000 EU	[46]

^1^ UHT milk, Ultra High Temperature treated milk, ^2^ SCC: somatic cell count.

**Table 2:** Effects of endotoxin on health.

Model	Strain/Dose	Administration	Exposure Time	Organ	Outcome	References
Infant rat	*Escherichia coli* 0127:B8 250–500 μg/kg/day (2,500,000–5,000,000 EU/day)	Oral gavage	6 days	Intestinal Plasma Lung Liver	Slow physical development, inflammation of intestinal tissue, increased TNF-α in plasma and lung, and increased CINC-1 in plasma, liver, lung, and distal small intestine	[39]
Mice	LPS (O111:B4) 300 μg/kg (3,000,000 EU/kg)	Oral gavage	2 h	Intestinal	LPS increased anxiety-like and decreased repetitive behaviors in wild type (WT) mice of both sexes.	[40]
Infant rats	*Escherichia coli* 0127:B8 250 μg/kg/day (2,500,000 EU/kg/day)	Oral gavage	6 days	Intestinal	Intestinal malformation; CINC mRNA secretion increased	[38]
Broiler Chickens	*Escherichia coli* 055:B5 2000 μg/kg (20,000,000 EU/kg)	Oral gavage	10 h	Intestinal	IL-6, IL-1β, and HSP70 increased; 3-OH C14 (part of LPS) increased	[76]
Mice	LPS (O111:B4) 3000 μg/kg (30,000,000 EU/kg)	Oral gavage	Twice a week	Intestinal	Abrogated the protection offered by gut microbiota eradication	[77]
Mice	*E. coli* O26:B6 2800 μg/kg (28,000,000 EU/kg)	Oral gavage	23 h	Chorda tympani nerve (CT)	Sensitivity to sweetness and saltiness was reduced	[78]
Rat	*E. coli* 0111:B4 300 μg/kg (3,000,000 EU/kg)	Injected	5 days	Intestinal	The presence of intestinal oxidative stress and increased intestinal permeability	[11]
Mice	LPS (not described) 5000 μg/kg (50,000,000 EU/kg)	Posterior pharyngeal instillation	1 h	Lung	Alveolar epithelial cell injury and increased vascular permeability; vascular endothelial growth factor receptor (VEGF/VEGFR) and TLR4/NF-κB pathways are involved in the development of LPS-stimulated ALI.	[79]
Human	*Salmonella abortus equi* endotoxin (0.8 ng/kg) (8 EU/kg)	Injected	4 h	Intestinal	Secretion of TNF-α and IL-6 and anorexia response	[80]
Mice	EtoH + LPS 2000 μg/kg (20,000,000 EU/kg)	Injected	6 h	Liver	Liver injury	[81]
Human Mice	*E. coli* O:113 LPS: 2 ng/kg (20 EU/kg) *E. coli* 055:B5 LPS: 5000 μg/animal (50,000,000 EU/animal)	Injected Oral load	0, 3, 6, 12, and 24 h 0, 3, and 6 h	Blood Ileum	Glucagon-like peptide 1 (GLP-1) and Toll-like receptor 4 (TLR4) increased	[82]
Mice	*E. coli* extract LPS 8 μg/kg (80,000 EU/kg)	Injected	5 days	Intestinal Blood	Memory impairment and colitis, and increased the absorption of orally administered LPS into the blood	[4]
Rat	LPS 200 μg/kg (2,000,000 EU/kg)	Injected	4 h	Blood	MIP-1 α, IL-10, MCP-1, IP-10, fractalkine, and TNF-α were increased, but there was no sign of fever	[83]
Rat	*E. coli* 055: B4 LPS 20 mg/kg (200,000,000 EU/kg)	Oral gavage	24 h	Intestinal	Inflammatory factor expression and intestinal epithelial damage	[84]

**Table 3:** Substances that inhibit endotoxin toxicity.

	Model	Strain/Dose	Therapeutic Dose	Outcome	References
Nutrients	Mice	Metabolic syndrome	Intestinal AP	Inhibited the absorption of endotoxin (LPS) induced by dietary fat	[119]
	Infant	Infant cardiopulmonary bypass (CPB)	Human liver AP	Reduced the harmful effects of endotoxemia following infant CPB	[137]
	Rats	*E. coli* 055: B4 LPS 20 mg/kg	Lactoferrin	Serum levels of TNF-α and IL-6 were significantly decreased	[84]
	Infant rat	Intestinal inflammation	Glutamine (Gln)	Endotoxin-induced intestinal inflammatory response was reduced	[38]
Probiotics	Rats	LPS 5 mg/kg	*Bifidobacterium infantis*	Increased IGF-1 expression and enhanced intestinal immune barrier function in endotoxin injured rats	[138]
	Mice	LPS isolated from *E. coli*	*Lactobacillus johnsonii* (LJ)	Reduced the levels of intestinal microflora and LPS in blood and alleviated memory impairment and colitis caused by TNBS and EC	[4]
Drugs/treatments	Mice	LPS (not described) 5000 μg/kg	20 mg/kg SU5416 + BW solution in DMSO	Inhibition of VEGF/VEGFR and TLR4/NF-κB signaling	[79]
	Mice	LPS (not described)	Resolvin E1; 24 h	Synthesis of alkaline phosphatase (ALP) to relieve endotoxin toxicity	[139]
	Rats	LPS (20 mg/kg)	Ketamine	LPS-induced gastric effusion and iNOS expression in the stomach and ileum were decreased	[97]
	SD rat	*E. coli* 055: B5 LPS 15 mg/kg	Salidroside (Sal)	Inhibition of iNOS, COX-2, NF-κB, and PI 3K/Akt/mTOR pathway/protection of heart from endotoxin	[140]
	Human	Endotoxin shock	endotoxin adsorption method (PMX-DHP)	Decreased procalcitonin (PCT) and endotoxin in blood	[141]

## Data Availability

Not applicable.
